# Comparative age-period-cohort analysis

**DOI:** 10.1186/s12874-023-02039-8

**Published:** 2023-10-18

**Authors:** Philip S. Rosenberg, Adalberto Miranda-Filho, David C. Whiteman

**Affiliations:** 1 Division of Cancer Epidemiology and Genetics, Biostatistics Branch, National Cancer Institute, NCI Shady Grove, Room 7E-130, 9609 Medical Center Drive, Bethesda, MD 20892 USA; 2 Cancer Control Group, QIMR Berghofer Medical Research Institute, Brisbane, QLD Australia

**Keywords:** Age-period-cohort model, Lexis diagram, Cancer surveillance research, SEER program

## Abstract

**Background:**

Cancer surveillance researchers analyze incidence or mortality rates jointly indexed by age group and calendar period using age-period-cohort models. Many studies consider age- and period-specific rates in two or more strata defined by sex, race/ethnicity, etc. A comprehensive characterization of trends and patterns within each stratum can be obtained using age-period-cohort (APC) estimable functions (EF). However, currently available approaches for joint analysis and synthesis of EF are limited.

**Methods:**

We develop a new method called Comparative Age-Period-Cohort Analysis to quantify similarities and differences of EF across strata. Comparative Analysis identifies whether the stratum-specific hazard rates are *proportional* by age, period, or cohort.

**Results:**

Proportionality imposes natural constraints on the EF that can be exploited to gain efficiency and simplify the interpretation of the data. Comparative Analysis can also identify differences or diversity in proportional relationships between subsets of strata (“pattern heterogeneity”). We present three examples using cancer incidence from the United States Surveillance, Epidemiology, and End Results Program: non-malignant meningioma by sex; multiple myeloma among men stratified by race/ethnicity; and in situ melanoma by anatomic site among white women.

**Conclusions:**

For studies of cancer rates with from two through to around 10 strata, which covers many outstanding questions in cancer surveillance research, our new method provides a comprehensive, coherent, and reproducible approach for joint analysis and synthesis of age-period-cohort estimable functions.

**Supplementary Information:**

The online version contains supplementary material available at 10.1186/s12874-023-02039-8.

## Introduction

In cancer surveillance research [1], a basic unit of analysis is a matrix of incidence or mortality hazard rates jointly indexed by age group and calendar period [2]. Alongside classical [3, 4] and contemporary [5–8] descriptive methods, the age-period-cohort (APC) model provides an established paradigm to quantify rate patterns and trends along each temporal direction – age, period, and birth cohort – adjusted for the other two [9].

There are several formulations of the age-period-cohort model for cancer research [10]. Here, we will focus on estimable functions (EF) of the parameters in an extended version [11] of Holford’s classic model [12]. EF are linear combinations of model parameters that are *invariant* with respect to identifiability constraints imposed on the model parameters to account for co-linearity between year of birth, year of event and age at event.

A comprehensive set of EF are available [13] based on the extended age-period-cohort model [11] (henceforth, the “New Model”). Amongst them, Local Drifts (LD) [14–17] and Cohort Rate Ratios (CRR) [18–20], are especially useful. For example, LD and CRR curves for colorectal cancer [14] provided critical evidence that prompted the ACS [21], the USPSTF [22], and the MSTF [23] to reevaluate the evidence and recommend that individuals in the United States at average risk begin colorectal cancer screening at age 45, down from age 50.

In practice, few studies examine a single rate matrix in isolation. Typically, hypotheses are explored by examining multiple sets of rates (strata) defined by sex, race/ethnicity, geographic region, tumor characteristics, etc [24–28]. Even so, currently available methods are limited to quantify similarities and differences of EF across strata.

Riebler et al. [29–31] considered stratified APC models with common age effects and smoothing priors on the second differences of the period and cohort effects, with estimates obtained by Markov Chain Monte Carlo (MCMC) and integrated nested Laplace approximations. Reimers et al. [32] used standard Wald tests to compare identifiable APC trend parameters in separate models fitted to each stratum, while Chien et al. [6, 33] compared summary statistics obtained from Lexis diagrams smoothed using Bernstein polynomials and MCMC. Most studies to date have relied on purely descriptive comparisons, which makes it challenging for researchers to draw objective and reproducible conclusions.

Frequently, the number of relevant strata $G$ is around 10 or less. In this paper we present a novel approach to tackle these essential small $G$ problems, which include studies of sex differences, racial and ethnic disparities, regional differences, tumor heterogeneity, etc. We call our new approach Comparative Age-Period-Cohort Analysis (“Comparative Analysis”). This work generalizes previous results for two-hazard problems [34]. Comparative Analysis is now made possible by the New Model via its fundamental decomposition principle.

Our approach makes three key assumptions. First, the stratum-specific hazard rates are available over the same age groups and calendar periods. This is always so for data obtained from official cancer registries. Second, the hazard rates are statistically independent within and between strata. This is always a reasonable basis for analysis when the cases in each stratum are different people, for example, for cases within strata defined by sex, demographic subgroup, geographic region, etc. Third, when we fit a *separate* New Model to each stratum, no concerning lack of fit (LOF) is detectable. This is the most important assumption. Current methods to assess LOF include estimating over-dispersion parameters, comparing observed and fitted values, and examining residuals. In those cases where the LOF is notable, one remedy is to split the rate matrix into blocks within which the LOF is minimized. See Best et al. [35] for details.

Comparative Analysis provides a comprehensive, coherent, and reproducible characterization of similarities and differences of EF across two, three, or more strata, along with efficient (model-based) estimates of EF and EF differences, including Local Drifts. It does so by identifying whether the stratum-specific hazard rates are *proportional* along one of the three fundamental temporal directions (age, period, or birth cohort). As we will show, when proportionality exists, it imposes natural constraints on the EF that can be exploited to gain efficiency and simplify the interpretation of the data.

Comparative Analysis can be conducted using a “hypothesis testing” approach or an “exploratory” approach. In the former, we aim to characterize proportionality across all the strata. In the latter, we don’t know a priori which stratum in the set – if any – might have rates that vary in concert. Therefore, our aim is to describe *pattern heterogeneity*. This can be accomplished by modeling the rates within *partitions* of the strata.

We will illustrate both approaches using data from the United States Surveillance, Epidemiology, and End Results (SEER) Program [36].

## Data

### SEER cancer incidence

We present three examples: 1) non-malignant meningioma by sex; 2) multiple myeloma among men stratified by race/ethnicity; 3) In situ melanoma by site among non-Hispanic white women. In our analyses race/ethnic groups are non-Hispanic white (NHW), non-Hispanic black (NHB), Hispanic (HIS) and Asian and Pacific Islander (API). Melanoma sites are head and neck (HN), upper limb (UL), trunk (Tr) and lower limb (LL). See Online Supplement Part 1 for details.

### Canonical case: a two-hazard problem

**Fig. 1:**
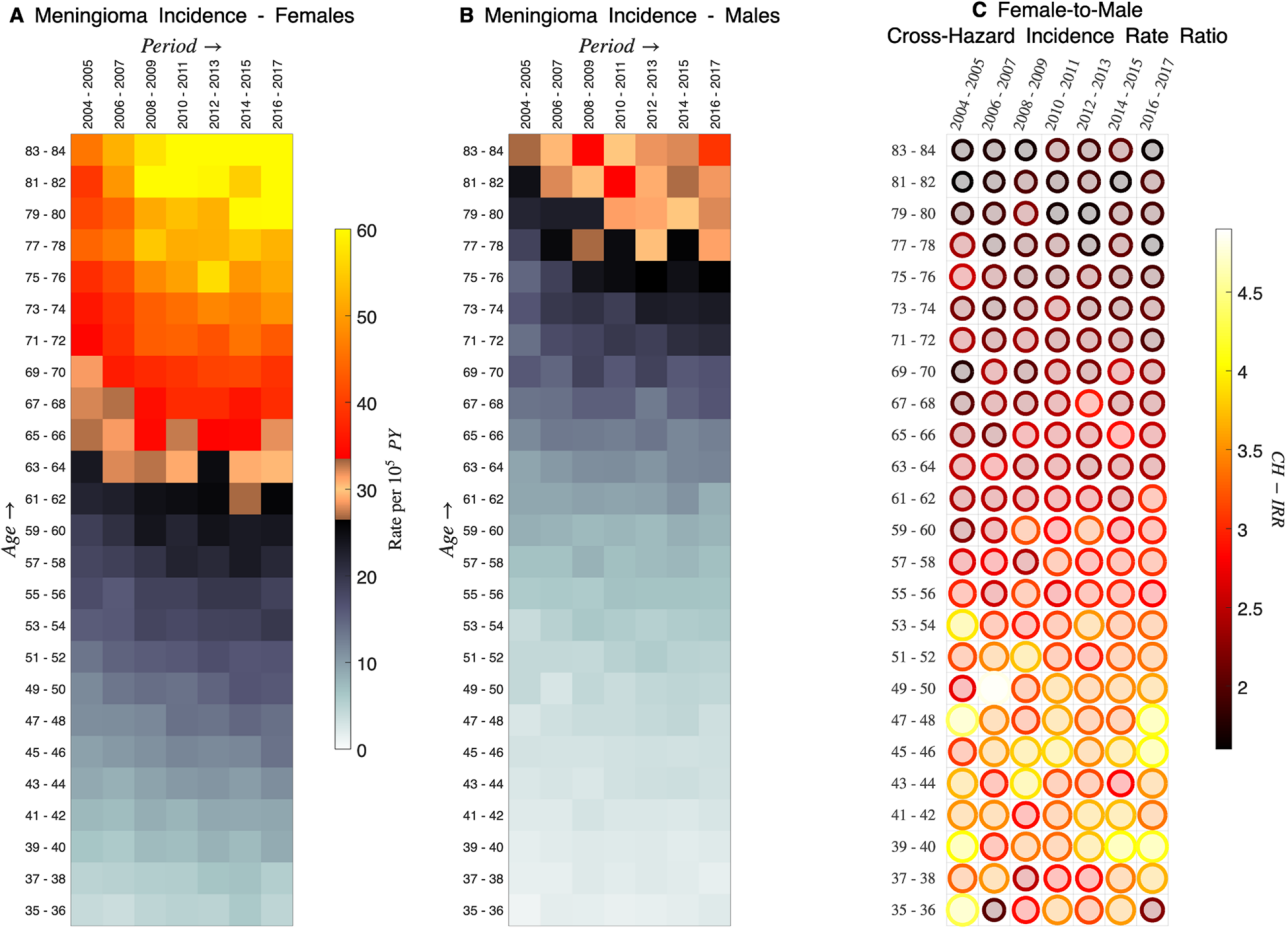
Meningioma incidence. Lexis diagram heat maps for meningioma incidence among NHW women (**A**) and men (**B**). See Online Supplement Part A for details. Inside colorbar shows color-mapped rates per 100,000 person-years. Bubble plot shows corresponding female-to-male cross-hazard incidence rate ratios (CH-RRs, **C**). CH-RR values are denoted by area and color (outside colorbar)

Figure 1 presents Lexis diagram heat maps for meningioma incidence among NHW women (Panel A) and men (Panel B). The heat maps reflect something we already know – meningioma incidence is higher among women. More revealing, the corresponding female-to-male *Cross-Hazard Rate Ratios* (CH-RRs, Panel C, bubble plot) show that the female excess is mostly constant over time (i.e., across the rows) but increases with decreasing age (i.e., down the columns).

### A four-hazard problem: multiple myeloma

**Fig. 2:**
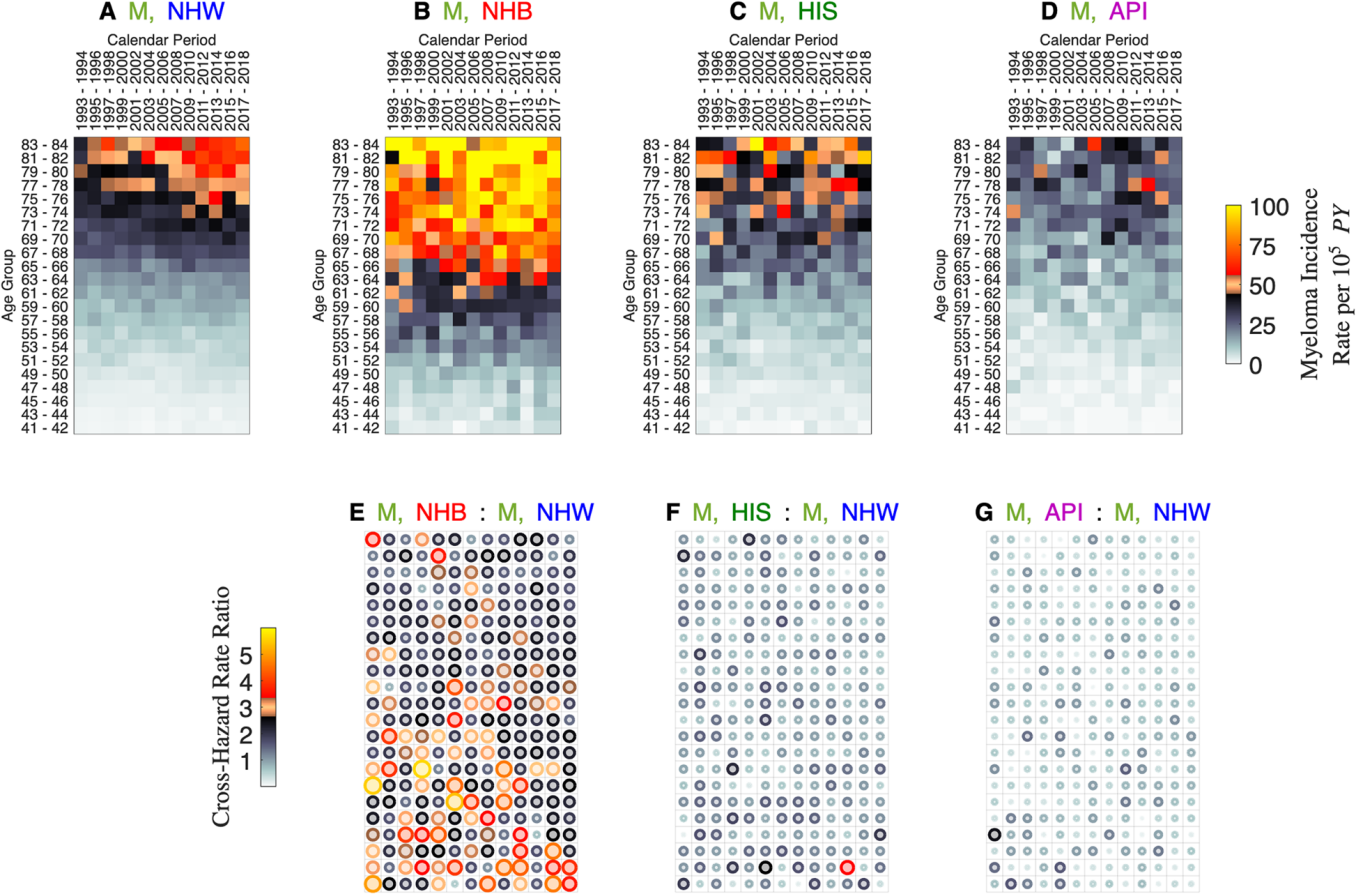
Myeloma incidence. Lexis diagram heat maps for myeloma incidence among men by race/ethnicity. Non-Hispanic Whites (NHW, **A**), Non-Hispanic Blacks (NHB, **B**); Hispanics (HIS, **C**), and Asians and Pacific Islanders (API, **D**). Bubble plots show corresponding CH-RRs for NHB versus NHW (**E**), HIS versus NHW (**F**), and API versus NHW (**G**). See Online Supplement Part A and the legend to Fig. 1 for details

Figure 2 presents Lexis diagram heat maps for multiple myeloma incidence among men within four race/ethnic groups (Panels A – D). The heat maps reflect that myeloma incidence is highest in NHB and lowest in API. Compared to NHW, corresponding CH-RRs for NHB versus NHW (Panel E), HIS versus NHW (Panel F), and API versus NHW (Panel G) are more-or-less constant. The excess in NHB versus NHW does appear highest in younger age groups (Panel E).

### Exploratory analysis: melanoma

As we will show in “Site differences of in situ melanoma” section, exploratory Comparative Analysis can reveal structure that is difficult to discern using traditional approaches.

## Methods

### Cross-hazard rate ratios: four canonical proportionalities

Comparative Analysis seeks to identify *proportional hazards* (PH) between strata along one time scale or another. For the case $G=2$, this problem was solved [34].

**Fig. 3:**
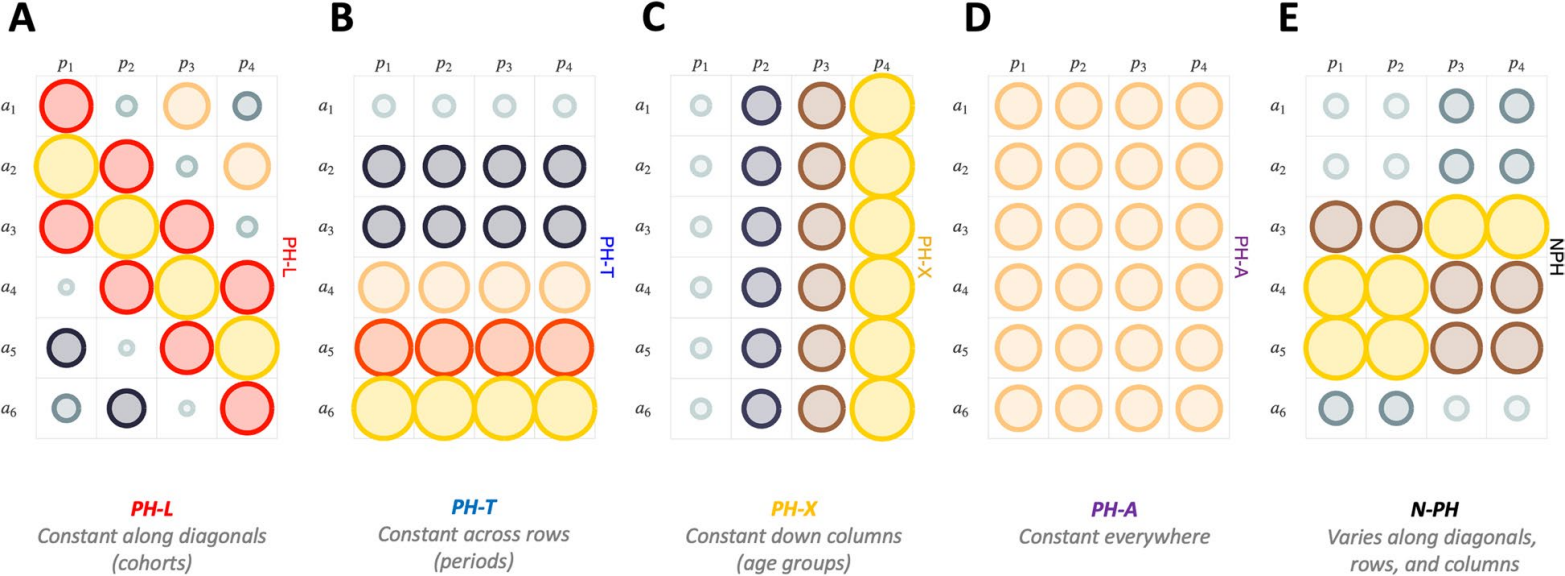
Proportional hazards: a schematic illustration. Grid represent a Lexis diagram indexed by age groups (rows) and calendar periods (columns). Area and color of each bubble maps CH-RR values for two sets of rates. PH-L, constant along diagonals or cohorts (**A**). PH-T, constant across rows or periods (**B**). PH-X, constant down columns or age groups (**C**). PH-A, constant everywhere (**D**). N-PH, not constant along diagonals, rows, and columns (**E**)

As illustrated in Fig. 3, when we compare two sets of hazard rates ascertained over the same Lexis diagram, each following an age-period-cohort model, there are five possibilities. The expected CH-RRs can be constant along diagonals (Panel A, “PH-L”, L for longitudinal), constant across rows (Panel B, “PH-T”, T for time), constant down the columns (Panel C, “PH-X”, X for cross-sectional age), or constant everywhere (Panel D, “PH-A”, A for absolute). Alternatively, if none of the PH models hold, then the CH-RRs are free to vary along diagonals, rows, and columns. When this happens, we say the data are not PH (Panel E, “N-PH"). Furthermore, it turns out that if any two of the PH models hold for a pair of hazards, then the third PH model must also hold; this is why there are five possible PH models rather than eight.

These result for $G=2$ were worked out using the algebra of the classic age-period-cohort model. Happily, using the algebra of the New Model, it is straightforward to generalize from $G=2$ to $G\ge 2$ and obtain useful formulas. Hence, we can now identify for the first time whether the scenarios shown in Fig. 3 hold *simultaneously* for all pairs of hazard rates within a larger ensemble of $G>2$ stratum and quantify the implications.

### A fundamental theorem for comparative age-period-cohort analysis

The New Model allows us to decompose age-period-cohort fitted rates four equivalent ways [11]. Each EF-based decomposition of the hazard rates ${\lambda }^{\left(g\right)}\left({a}_{*},{p}_{*},{c}_{*}\right),g=1,\dots ,G$ includes a baseline hazard function, a main effect, and an interaction along one of the three temporal directions of age ${a}_{*}$, period ${p}_{*}$ or cohort ${c}_{*}={p}_{*}-{a}_{*}$. These decompositions can be related to the canonical proportional hazards models illustrated in Fig. 3. For a definition of each EF in terms of the APC model parameters, please refer to Table 1 of Rosenberg [11].

**Table 1:**
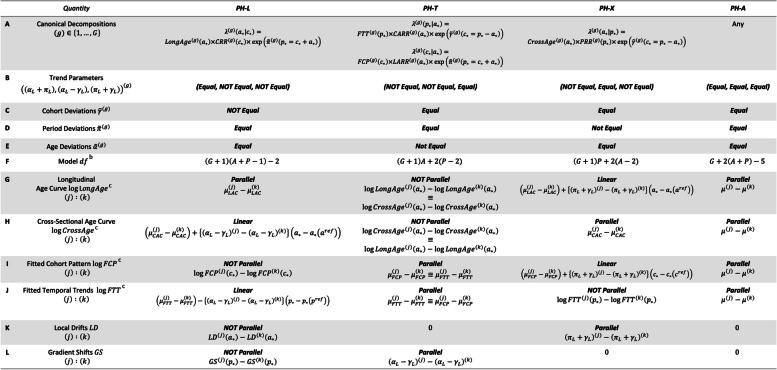
Comparative age-period-cohort analysis^a^

^a^ Set-up for $G\ge 2$ stratum each defined on the same Lexis diagram with $A$ age groups and $P$ calendar periods

^b^ Model $df$ for *N-PH* is $2G\left(A+P-2\right)$

^c^ Intercept terms for log-scale estimable functions are: $\mu_{FTT}^{(k)}=\log CrossAge^{(k)}\left(a_\ast\right)$, ${\mu }_{CAC}^{(k)}=\mathrm{log}{FTT}^{(k)}\left({p}_{*}\left({p}_{ref}\right)\right)$, $\mu_{LAC}^{(k)}=\log{FCP}^{(k)}\left(c_\ast\left(c_{ref}\right)\right)$, $\mu_{FCP}^{(k)}=\log{LongAge}^{(k)}\left(a_\ast\left(a_{ref}\right)\right)$

#### PH-L: Longitudinal in age

Given the decompositions:

$$\begin{array}{c}{\lambda }^{(g)}\left({a}_{*}|{c}_{*}\right)={LongAge}^{(g)}\left({a}_{*}\right)\times {CRR}^{(g)}\left({c}_{*}\right)\times \mathrm{exp}\left({\widetilde{\pi }}^{(g)}\left({p}_{*}={c}_{*}+{a}_{*}\right)\right),g=1,\dots ,G\end{array}$$

If the stratum-specific Longitudinal Age Curves ${LongAge}^{(g)}\left({a}_{*}\right)$ are proportional to each other, the period deviations ${\widetilde{\pi }}^{\left(g\right)}\left({p}_{*}\right)$ are all equal, but the Cohort Rate Ratios ${CRR}^{(g)}\left({c}_{*}\right)$ are *not* equal for any two or more stratum, we say the rates are PH-L. When PH-L holds, CH-RRs are determined by birth cohort effects:

$$PH-L\Rightarrow{\frac{\lambda^{(j)}\left(a_\ast\vert c_\ast\right)}{\lambda^{(k)}\left(a_\ast\vert c_\ast\right)}=RR_{CH}^{\left(j\right):(k)}}\left(c_\ast\right)=\frac{{FCP}^{(j)}\left(c_\ast\right)}{{FCP}^{(k)}\left(c_\ast\right)}$$

In this expression, ${FCP}^{(g)}\left({c}_{*}\right)$ is an EF called the Fitted Cohort Pattern that describes the expected rate at an arbitrary reference age ${a}_{*}({a}_{ref})$ for each birth cohort in stratum $(g)$.

This expression works because ${CRR}^{(g)}\left({c}_{*}\right)\equiv \frac{{FCP}^{\left(g\right)}\left({c}_{*}\right)}{{FCP}^{\left(g\right)}\left({c}_{*}\left({c}_{ref}\right)\right)}\equiv \frac{{FCP}^{\left(g\right)}\left({c}_{*}\right)}{{LongAge}^{\left(g\right)}\left({a}_{*}\left({a}_{ref}\right)\right)}$. That is, the intercept terms in the Longitudinal Age Curves cancel the reference values in the Cohort Rate Ratio curves, which permits the CH-RRs values to range freely. This is crucial: CH-RRs are not *referent* RRs, with one category arbitrarily chosen as a baseline. Rather, they are *floating* RRs. For example, in Fig. 1C, the CH-RRs never fall below 1.0.

#### PH-T: Cross-sectional in period

Given the decompositions:

$$\begin{array}{c}{\lambda }^{(g)}\left({p}_{*}|{a}_{*}\right)={FTT}^{(g)}\left({p}_{*}\right)\times {CARR}^{(g)}\left({a}_{*}\right)\times \mathrm{exp}\left({\widetilde{\gamma }}^{(g)}\left({c}_{*}={p}_{*}-{a}_{*}\right)\right)\end{array},g=1,\dots ,G$$

If the stratum-specific Fitted Temporal Trends ${FTT}^{(g)}\left({p}_{*}\right)$ are proportional, the cohort deviations ${\widetilde{\gamma }}^{\left(g\right)}\left({c}_{*}\right)$ are all equal, but the Cross-Sectional Age Rate Ratios ${CARR}^{(g)}\left({a}_{*}\right)$ are *not* equal for any two or more stratum, we say the rates are PH-T. When PH-T holds, CH-RRs are determined by age effects:

$$PH-T\Rightarrow {\frac{{\lambda }^{(j)}\left({p}_{*}|{a}_{*}\right)}{{\lambda }^{(k)}\left({p}_{*}|{a}_{*}\right)}=RR_{CH}^{\left(j\right):(k)}}\left({a}_{*}\right)=\frac{{CrossAge}^{(j)}\left({a}_{*}\right)}{{CrossAge}^{(k)}\left({a}_{*}\right)}$$

This works because ${CARR}^{(g)}\left({a}_{*}\right)\equiv \frac{{CrossAge}^{\left(g\right)}\left({a}_{*}\right)}{{CrossAge}^{\left(g\right)}\left({a}_{*}\left({a}_{ref}\right)\right)}\equiv \frac{{CrossAge}^{\left(g\right)}\left({a}_{*}\right)}{{FTT}^{(g)}\left({p}_{*}\left({p}_{ref}\right)\right)}$.

Hence, the intercept terms in the Fitted Temporal Trends cancel the reference values in the Cross-Sectional Age Rate Ratio curves, which permits the CH-RRs values to range freely.

#### PH-T: Cross-sectional in cohort

Given the decompositions:

$$\begin{array}{c}{\lambda }^{(g)}\left({c}_{*}|{a}_{*}\right)={FCP}^{(g)}\left({c}_{*}\right)\times {LARR}^{(g)}\left({a}_{*}\right)\times \mathrm{exp}\left({\widetilde{\pi }}^{(g)}\left({p}_{*}={c}_{*}+{a}_{*}\right)\right), g=1,\dots ,G\end{array}$$

If the stratum-specific Fitted Cohort Patterns ${FCP}^{(g)}\left({c}_{*}\right)$ are proportional, the period deviations ${\widetilde{\pi }}^{\left(g\right)}\left({p}_{*}={c}_{*}+{a}_{*}\right)$ are all equal, but the Longitudinal Age Rate Ratios ${LARR}^{(g)}\left({a}_{*}\right)$ are *not* equal for any two or more stratum, we also say the rates are PH-T. From these expressions, CH-RRs are also determined by age effects:

$$PH-T\Rightarrow {\frac{{\lambda }^{(j)}\left({c}_{*}|{a}_{*}\right)}{{\lambda }^{(k)}\left({c}_{*}|{a}_{*}\right)}=RR_{CH}^{\left(j\right):(k)}}\left({a}_{*}\right)=\frac{{LongAge}^{(j)}\left({a}_{*}\right)}{{LongAge}^{(k)}\left({a}_{*}\right)}$$

This works because ${LARR}^{(g)}\left({a}_{*}\right)\equiv \frac{{LongAge}^{(g)}\left({a}_{*}\right)}{{LongAge}^{\left(g\right)}\left({a}_{*}\left({c}_{ref}\right)\right)}\equiv \frac{{LongAge}^{\left(g\right)}\left({a}_{*}\right)}{{FCP}^{(g)}\left({c}_{*}\left({c}_{ref}\right)\right)}$.

Hence, the intercept terms in the Fitted Cohort Patterns cancel the reference values in the Longitudinal Age Rate Ratio curves, which permits the CH-RRs values to range freely.

So now we have two different ways of getting PH-T. Fortunately, the results are equivalent.

#### Corollary 1

When PH-T holds,

$$\frac{{LongAge}^{(j)}\left({a}_{*}\right)}{{LongAge}^{(k)}\left({a}_{*}\right)}=\frac{{CrossAge}^{(j)}\left({a}_{*}\right)}{{CrossAge}^{(k)}\left({a}_{*}\right)}$$

For all pairs of strata $\left(j\right)$ and $\left(k\right)$. This holds because

$$\begin{array}{c}{LongAge}^{\left(j\right)}\left({a}_{*}\right)=\mathrm{exp}\left(\left\{{\left({\alpha }_{L}+{\pi }_{L}\right)}^{\left(j\right)}-{\left({\alpha }_{L}-{\gamma }_{L}\right)}^{\left(j\right)}\right\}\left({a}_{*}-{\overline{a} }_{*}\right)\right){CrossAge}^{\left(j\right)}\left({a}_{*}\right)\\ = \mathrm{exp}\left({\left({\pi }_{L}+{\gamma }_{L}\right)}^{\left(j\right)}\left({a}_{*}-{\overline{a} }_{*}\right)\right){CrossAge}^{\left(j\right)}\left({a}_{*}\right)\end{array}$$

Under PH-T, the Fitted Temporal Trends ${FTT}^{(g)}\left({p}_{*}\right)$ are all proportional, which implies that the Net Drifts $, {\left({\pi }_{L}+{\gamma }_{L}\right)}^{\left(g\right)}$ are all equal. Hence

$$\frac{{LongAge}^{(j)}\left({a}_{*}\right)}{{LongAge}^{(k)}\left({a}_{*}\right)}-\frac{{CrossAge}^{\left(j\right)}\left({a}_{*}\right)}{{CrossAge}^{\left(k\right)}\left({a}_{*}\right)}=0.$$

Furthermore, under PH-T, it follows that

$$\frac{{FCP}^{(j)}\left({c}_{*}\right)}{{FCP}^{(k)}\left({c}_{*}\right)}-\frac{{FTT}^{\left(j\right)}\left({p}_{*}\right)}{{FTT}^{\left(k\right)}\left({p}_{*}\right)}=0,$$

for all values of ${c}_{*}$ and ${p}_{*}$.

#### PH-X: Cross-sectional in age

Given the decompositions:

$$\begin{array}{c}{\lambda }^{(g)}\left({a}_{*}|{p}_{*}\right)={CrossAge}^{(g)}\left({a}_{*}\right)\times {PRR}^{(g)}\left({p}_{*}\right)\times \mathrm{exp}\left({\widetilde{\gamma }}^{(g)}\left({c}_{*}={p}_{*}-{a}_{*}\right)\right), g=1,\dots ,G\end{array}$$

If the stratum-specific Cross-Sectional Age Curves ${CrossAge}^{(g)}\left({p}_{*}\right)$ are proportional, the cohort deviations ${\widetilde{\gamma }}^{\left(g\right)}\left({c}_{*}\right)$ are all equal, but the Period Rate Ratio curves ${PRR}^{(g)}\left({p}_{*}\right)$ are *not* equal for any two or more stratum, we say the rates are PH-X. When PH-X holds, CH-RRs are determined by period effects:

$$PH-X\Rightarrow {\frac{{\lambda }^{(j)}\left({a}_{*}|{p}_{*}\right)}{{\lambda }^{(k)}\left({a}_{*}|{p}_{*}\right)}=RR_{CH}^{\left(j\right):(k)}}\left({p}_{*}\right)=\frac{{FTT}^{(j)}\left({p}_{*}\right)}{{FTT}^{(k)}\left({p}_{*}\right)}$$

This works because ${PRR}^{(g)}\left({p}_{*}\right)\equiv \frac{{FTT}^{(g)}\left({p}_{*}\right)}{{FTT}^{\left(g\right)}\left({p}_{*}\left({a}_{ref}\right)\right)}\equiv \frac{{FTT}^{\left(g\right)}\left({p}_{*}\right)}{{CrossAge}^{(g)}\left({a}_{*}\left({a}_{ref}\right)\right)}, g=1, \dots ,G$.

Hence, the intercept terms in the Cross-Sectional Age curves cancel the reference values in the Period Rate Ratio curves, which permits the CH-RRs values to range freely.

#### PH-A: Absolute proportionality

It is easy to demonstrate that if any two models PH-L, PH-T, or PH-X hold, then the third model must also hold. When this happens, we say the data are “absolutely proportional” (PH-A). Under PH-A the CH-RRs depend only on the intercepts, $PH-A\Rightarrow {RR}_{CH}^{\left(j\right):(k)}={e}^{\left({\mu }^{(j)}-{\mu }^{(k)}\right)}$.

#### N-PH: Not proportional

If none of the models PH-L, PH-T, or PH-X hold (which implies that PH-A cannot hold), we say the data are “not proportional” (N-PH). Under N-PH the CH-RRs vary freely according to age, period, and cohort.

#### Corollary 2

The results described below are summarized in Table 1. Online Supplement Part 2 discusses some computational details.

Recall that an LD curve is obtained by sliding a window of width $P$ (number of calendar periods) through the cohort deviations and extracting the least squares slope, and then adding these “deflection” terms to the overall Net Drift.

It follows that the LD are *not* parallel under PH-L or N-PH; *identical* under PH-T and PH-A; and *parallel* under PH-X (Table 1, Row K). Furthermore, under PH-X, the constant difference between LD curves for stratum $\left(j\right)$ versus $\left(k\right)$ is determined by the corresponding difference between the Net Drifts. Model-based LD curves are also more precise.

PH-T is characterized by constant ratios between the Fitted Temporal Trends (Table 1, Row J), and *identical* constant ratios between the Fitted Cohort Patterns (Table 1, Row I). Model-based estimates for these EF are also more precise than the corresponding unconstrained estimates obtained under the N-PH model.

Conversely, under PH-L, the Longitudinal Age Curves are all proportional (Table 1, Row G), but the cohort deviations (Table 1, Row C) are heterogeneous. However, because the period deviations are all equal (Table 1, Row D), differences between the Fitted Temporal Trends (Table 1, Row J) vary linearly and are estimated with increased precision.

#### Model selection

Each PH model can be evaluated for lack-of-fit via *P*-values. Even so, *P*-values don’t always tell the whole story. Information Theory provides a means to balance statistical significance with *predictive utility*; for this purpose, we will use the Bias-Corrected Akaike Information Criterion ($${AIC}_{c})$$, as recommend by Burnham and Anderson [37].

## Hypothesis-based comparative analysis: results

### Example 1: Sex differences in meningioma

**Fig. 4:**
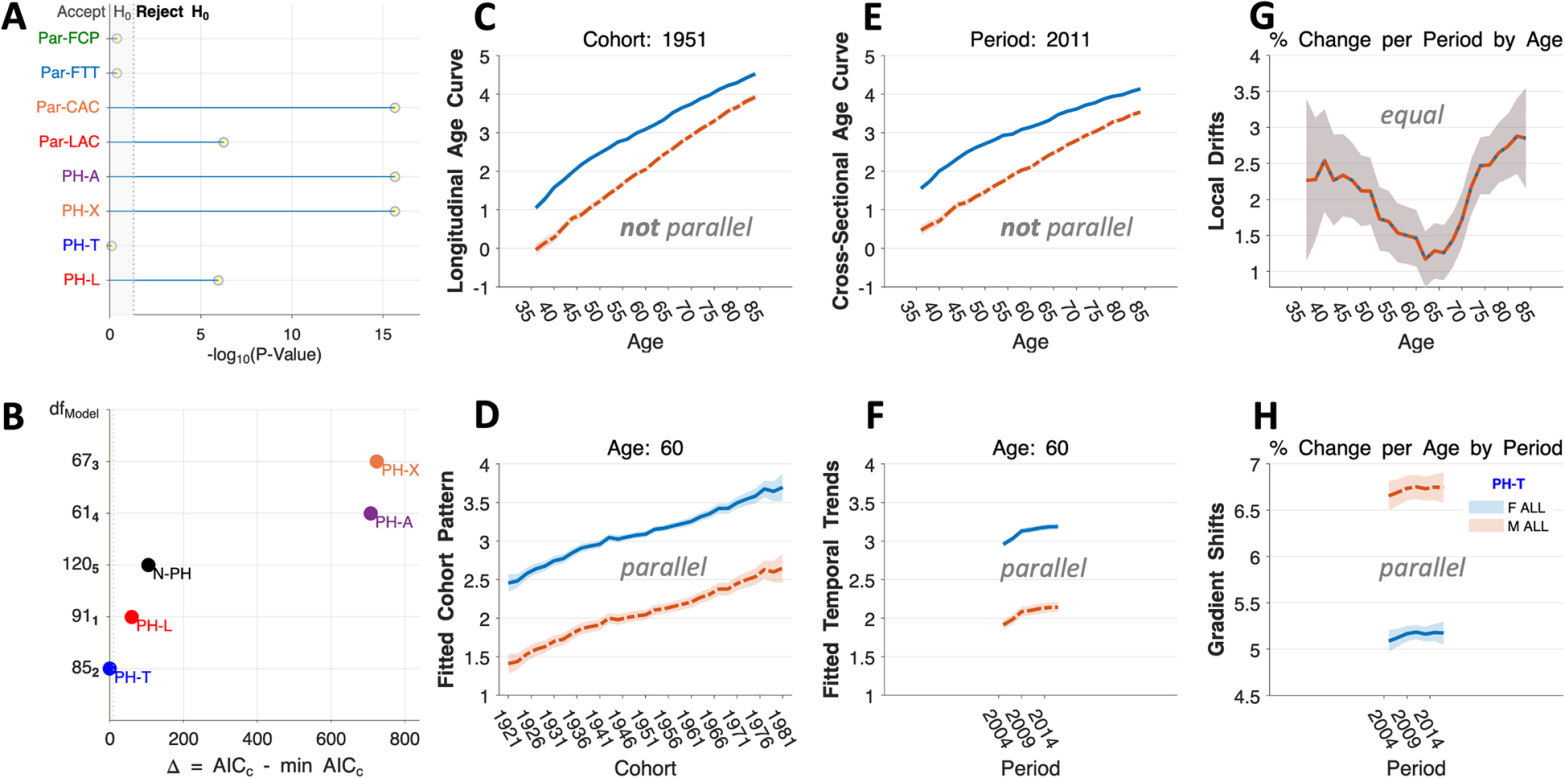
Meningioma incidence: model selection and estimable functions. **A** Composite goodness-of-fit tests [11] for proportionality (PH-L, PH-T, PH-X, PH-A) and EF parallelism (Par-LAC, parallel Longitudinal Age Curves; Par-CAC, parallel Cross-Sectional Age Curves; Par-FTT, parallel Fitted Temporal Tends; Par-FCP, parallel Fitted Cohort Patterns). Abscissa values show $-\log_{10}$ (*P *– Values). **B** Bias-corrected AIC values, ${AIC}_{c}$, relative to the model with lowest ${AIC}_{c}$. **C** – **H** Females, solid blue curves, Males, dashed red curves. Longitudinal Age Curves (**C**), Fitted Cohort Patterns (**D**), Cross-Sectional Age Curves (**E**), Fitted Temporal Trends (**F**), Local Drifts (**G**), and Gradient Shifts (**H**). All curves are estimated under the PH-T model. **C**, **D**, **E**, and **F** EF rates per 100,000 on the natural log scale. **G** and **H** EF annual percentage changes

Composite Tests for proportionality fail to reject PH-T, but strongly reject PH-L, PH-X, and PH-A (Fig. 4A). Therefore, based on *P*-values, the rates are PH-T. Furthermore, the ${AIC}_{c}$ is minimized for PH-T (Fig. 4B), and no other model comes close.

Figure 4 panels C – H present EF curves based on the PH-T model (females, solid blue; males, dashed red). The female excess narrows with age (Fig. 4C and E). Incidence is increasing in successive birth cohorts (Fig. 4D) but slowing over time (Fig. 4F) at the same rate in women and men. Incidence is increasing in every age group (Local Drifts, Fig. 4G). The U-shaped pattern in the Local Drifts reflects that the moderation that occurred among Baby Boomers was not sustained in younger birth cohorts (Fig. 4D). Increases over time in the younger cohorts may reflect increases in clinical detection activities (e.g., brain imaging) over time [38]. The Gradient Shifts are parallel and stable (Fig. 4H).

### Example 2: Race/ethnic differences in myeloma

**Fig. 5:**
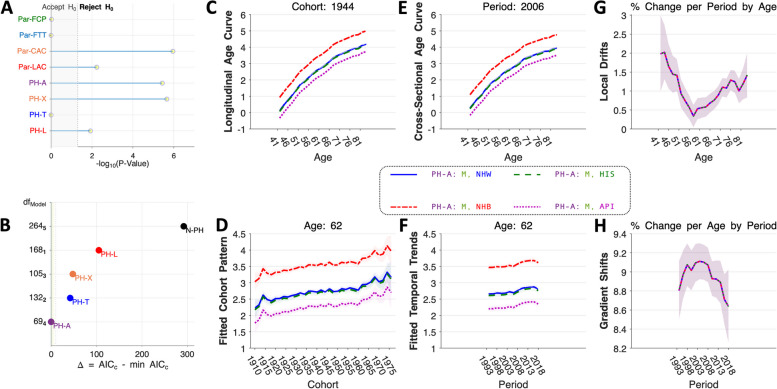
Myeloma incidence: model selection and estimable functions. See legend to Fig. 4 for details. NHW, solid blue, NHB, dot-dash red, HIS, dash Green, API, dotted magenta. All curves estimated under the PH-A model

The rates are PH-T based on *P*-values (Fig. 5A), but PH-A has the lowest ${AIC}_{c}$ (Fig. 5B). Indeed, neither the PH-T model nor any other model comes close. EF curves based on the PH-A model are shown in Fig. 5 panels C – H. Incidence by age is highest among NHB, lowest among API, and nearly identical among NHW and HIS (Fig. 5C and E). Under PH-A, the LD curves do not differ by race/ethnicity (Fig. 5G). The gradient shifts (Fig. 5H) are equal with an inverted U-shape. Incidence over time is increasing most rapidly in the youngest age groups with the same annual percentage changes in each race/ethnic group. Based on the PH-A model parameters, Myeloma incidence is consistently 2.24-fold (95% Confidence Interval [CI]: 2.2 – 2.3) higher in NHB versus NHW; marginally lower (CH-RR = 0.95, 95% CI: 0.91 – 0.99) in HIS versus NHW, and 0.37-fold (95% CI: 0.33 – 0.40) lower in API versus NHW (based on CH-RR values of 0.63 (95% CI: 0.60 – 0.66).

## Pattern heterogeneity

Epidemiologists say that “patterns” are heterogeneous when two or more parameters vary across stratum. This is distinct from generic heterogeneity of a single parameter. There are many notable examples of pattern heterogeneity in the literature [39–44].

In the setting of a Comparative Analysis, we define *Pattern Heterogeneity* as the occurrence of differences or diversity in proportional relationships (as determined by multiple parameters, Table 1) between subsets of strata. We can identify Pattern Heterogeneity by modeling *partitions* of the strata and ranking the partitions by predictive utility, as measured by the bias-corrected AIC for the partition. We will call this process a *Multiplex Analysis* because the algorithm can be parallelized.

For example, suppose we have $G=4$ stratum $A,B,C,\text{ and} D$. A partition is a division of the strata into non-overlapping subsets, e.g.,

$$\left\{ABCD\right\}, \left\{A\}|\{BCD\right\}, \left\{B\}|\{ACD\right\},\dots ,\left\{AB\}|\{CD\right\},\dots ,\{AB\}|\left\{C\right\}\left\{D\right\}, \dots , \{A\}|\{B\}|\{C\}|\{D\}$$

Call the set of non-empty subsets ${\mathcal{S}}_{G}=\left\{{\mathcal{s}}_{1},{\mathcal{s}}_{2},\dots ,{\mathcal{s}}_{N}\right\}$ and the corresponding set of partitions ${{P}}_{G}=\left\{{{p}}_{1},{{p}}_{2},\dots ,{{p}}_{B}\right\}$. For $G=4$, $N=15$ and $B=15$. The number of partitions $B$ is described by Bell’s numbers [45], and efficient algorithms are available to enumerate the partitions [46, 47].

Bell’s numbers increase exponentially: The first 10 are $B=1,\;2,\;5,\;15,\;52,\;203,\;877,\;4140,\;21147,\;\mathrm{and}\;115975$.

To determine the bias-corrected AIC value for each partition ${{p}}_{b},b=\mathrm{1,2},\dots ,B$ we need to keep track of its constituent subsets in ${\mathcal{S}}_{G}$ and the order of occurrence of those subsets. Record these values in a $B\times N$ *correspondence matrix* ${T}_{G}$.

**Figure:**
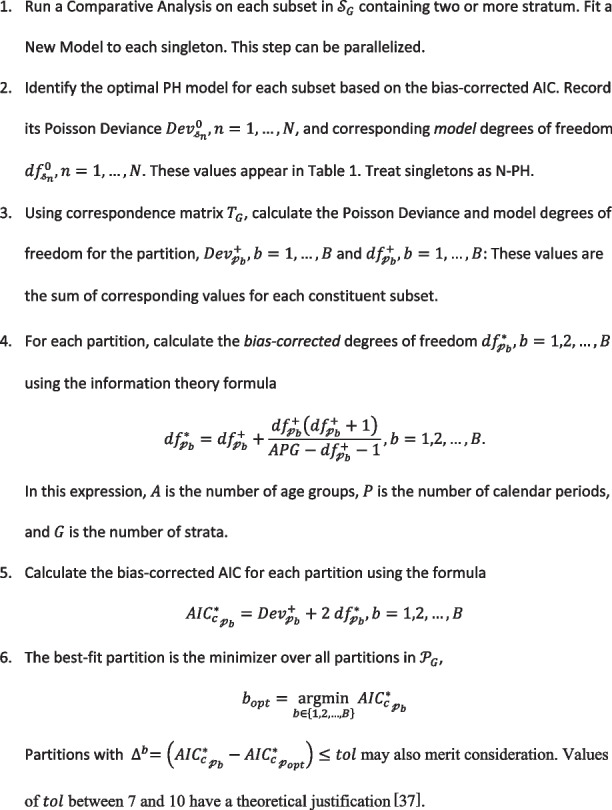
**Algorithm 1.** Multiplex analysis [37]

## Exploratory comparative analysis: example

### Site differences of in situ melanoma

**Fig. 6:**
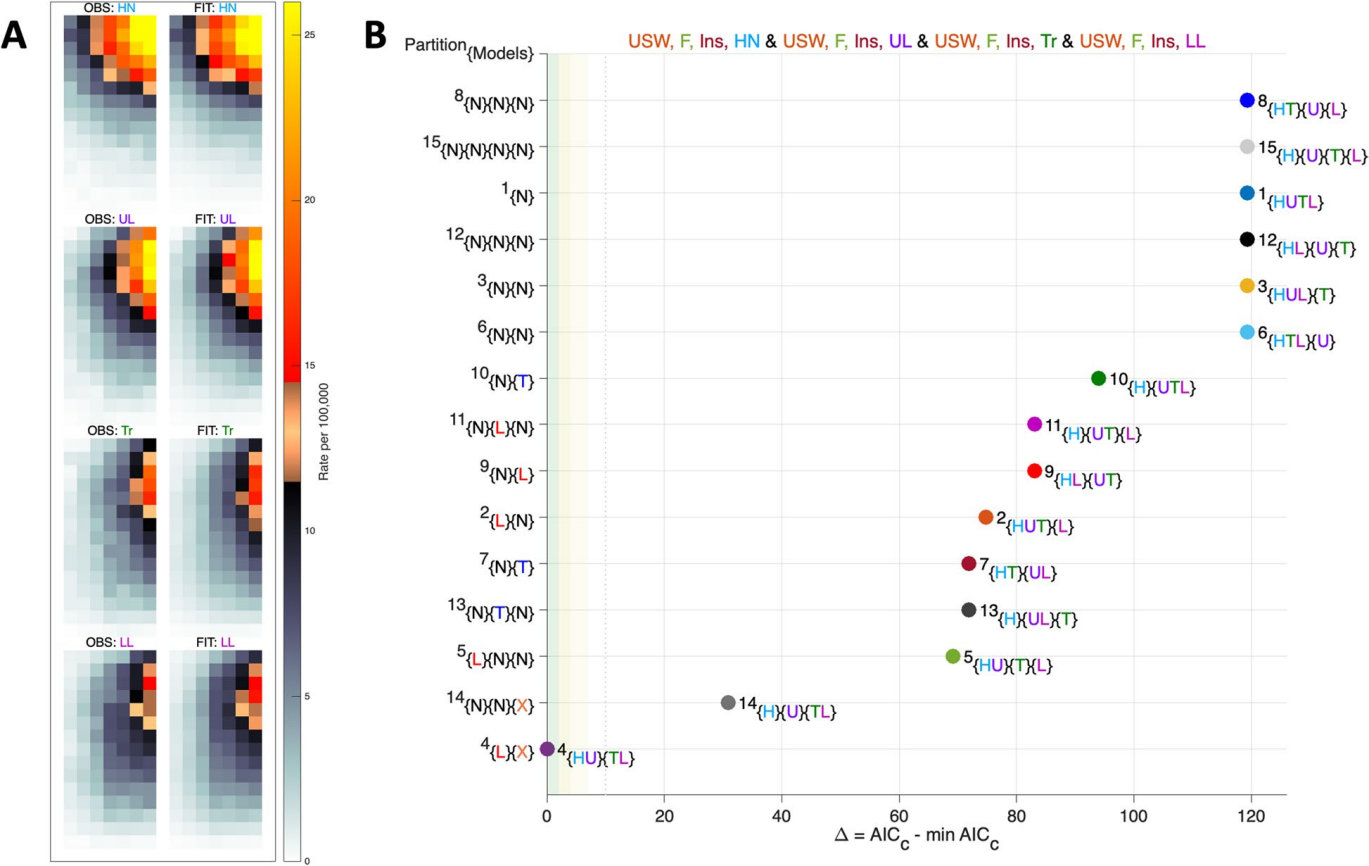
NHW female melanoma incidence by site: multiplex analysis. **A** Heat maps of observed data (left column) and optimal fitted values (right column). **B** Model selection. Abscissa values ${\Delta }^{b}=\left({{AIC}_{c\ p}^{*}}_{_{b}}-{{AIC}_{c\ p}^{*}}_{_{opt}}\right),b=1,\dots ,15$, differentials of bias-corrected ${AIC}_{c}$ for the 15 partitions of 4 strata, versus the overall minimum. Body site codes are Head and Neck, blue “H”, Upper Limb, purple “U”, Trunk, green “T”, and Lower Limb, pink “L”. The optimal partition number is 4, which consists of subsets {*HU*}{*TL*}. Y-axis Label {*L*}{*X*} indicates that subset {*HU*} is PH-L and subset {*TL*} is PH-X

Observed data are shown in Fig. 6A (left column). Results of a Multiplex Analysis are summarized in Fig. 6B. The best-fit partition which appears in the lower-left corner of the plot in Panel B identifies pattern heterogeneity: head and neck (H) and upper limb (U) are PH-L, whereas trunk (T) and lower limb (L) are PH-X. No other partition fits as well. Fitted values for this partition are shown in Fig. 6A (right column). The fitted values are very similar to the observed values.

**Fig. 7:**
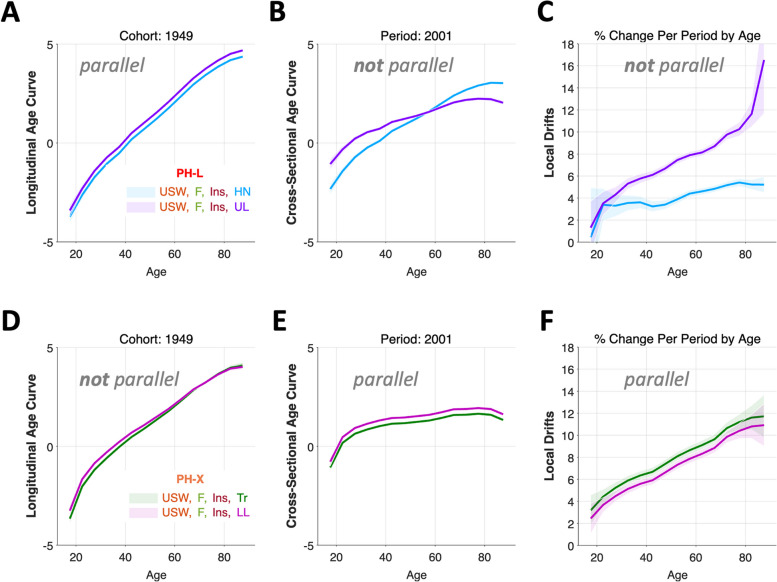
Melanoma incidence: estimable functions. **A**-**C** PH-L model results for Head and Neck (HN) and Upper Limbs (UL). **D**-**E** PH-X model results for Trunk (Tr) and Lower Limbs (LL). **A**, **B**, **D**, and **E** Longitudinal and Cross-Sectional Age Curves. Rates per 100,000 on the natural log scale. **C** and **F** Local Drift annual percentage changes

EF curves for this configuration are shown in Fig. 7. For head and neck (HN) and upper limb (UL), the *Longitudinal* Age Curves are parallel (Panel A), whereas the Cross-Sectional Age Curves and Local Drifts are not (Panels B and C, respectively). Indeed, whereas incidence of HN is increasing at a qualitatively similar annual percentage change over time in all age groups, with increasing age, UL is increasing much more quickly than HN over time. In contrast, for trunk (Tr) and lower limb (LL), the Cross-Sectional Age Curves (Panel E) and the Local Drifts (Panel F) are parallel, but the Longitudinal Age Curves are not (Panel D). In every age group, Tr increased by 0.7 (95% CI: 0.30 – 1.15) percent per calendar year faster than LL.

## Discussion

In practice, comparative studies can be surprisingly hard. This reflects the multivariate nature of the problem. Each stratum-specific rate matrix spans four timescales, each with informative EF, and there are four relevant cross-hazard proportionalities. Our new methods automate and streamline the identification of key similarities and differences between stratum within a comprehensive framework. In this regard, we believe the summary of our results for hypothesis-based comparative studies in Table 1 provides a helpful overview for the practitioner.

In brief, under PH-L the age-associated natural history curves in each stratum are parallel, but the cohort and period effects are not. In contrast, under PH-T, the natural histories are distinct, but the cohort and period effects are parallel. Under PH-X, differences between strata are modulated through the Net Drifts and period deviations. Under PH-A, stratum-specific event rates differ only by constants.

As illustrated by our examples, our approach can provide new insights. For meningioma, the female excess narrows with age, but temporal patterns are strikingly similar over time and across generations – a textbook example of PH-T. For male myeloma, the disparity among black men has long been recognized, but the absolute proportionality of the rates across race/ethnic groups has not. For melanoma in NHW females, our exploratory approach identified proportional Longitudinal Age Curves and distinct Local Drifts for HN and UL, versus distinct Longitudinal Age Curves and proportional Local Drifts for Tr and LL. In our experience, our examples are typical, not outliers. In ongoing studies of other cancers, proportionality – with and without pattern heterogeneity – is a common occurrence. These findings provide new clues for cancer researchers and medical decision makers to follow.

In our view, the reasons for these successes are 1) our method’s reliance on information theory, specifically, the bias-corrected AIC statistic, to drive the model selection process, 2) the New Model often provides an excellent second-order approximation to the rates in each stratum, and 3) the proportionality relationships that the method is designed to detect, i.e., PH-L, PH-T, PH-X or PH-A (Fig. 3), make the most sense from an epidemiologic perspective.

Our exploratory approach builds upon the foundation provided by our hypothesis-based approach. Indeed, within any given subset of a partition, if a PH model holds (more or less, given the limitations discussed below), it’s a win–win-win: It simplifies the story; it identifies which EF drives cross-hazard heterogeneity; it provides increased precision. In contrast, if the rates are N-PH, then one can conclude that the rates are *undeniably* heterogeneous. In that case, all the EF contribute to the cross-hazard differences, and any description of the data should make note of this fact.

Our approach has several limitations. The famous aphorism “all models are wrong, but some are useful [48]” describes our approach to a “T”. The New Model, which provides the foundation for both hypothesis-based and exploratory comparative analysis, can never be *entirely* correct. Furthermore, it is naïve to assume that a PH model could *flawlessly* characterize relationships between strata. Occasionally, more than one PH model or partition may have similar bias-corrected AIC values. When that happens, the fitted values are similar, but it remains unclear which model or partition provides the most robust insight. In this situation, one could employ model averaging [37].

With these limitations in mind, Algorithm 1 for Multiplex Analysis can readily be performed for $2\le G\le 8$ strata. Because the algorithm can be parallelized (see Online Supplement Part 2), it appears feasible for slightly larger values of $G$. Even so, the complexity of the analysis increases exponentially with $G$, and for $G=12$, the number of partitions exceeds 4.2 million. At some point, one must restrict the number of strata or evaluated partitions. As illustrated here, many important problems involve fewer than 10 strata. Furthermore, adding more strata isn’t necessarily better, because the bias correction term increases as the square of the number of added parameters, which tends to make our methods less sensitive as $G$ increases.

Bayesian methods are attractive when $G>10$ [49–51]. Bayesian analysis can estimate the distribution of EF across an arbitrary number of strata assuming that the parameters are realizations from an estimated posterior distribution. This approach implies that the parameters are broadly similar. In contrast, a Multiplex Analysis of $2\le G\le 10$ strata in search of pattern heterogeneity does not make the same assumption. Perhaps hybrid multi-scale methods could be developed that marry the strengths of each approach. In the context of a spatial age-period-cohort analysis, regions could be partitioned using Multiplex Analysis, and small areas within related regions could be modeled using Bayesian methods.

Another complementary approach for small $G$ problems is to smooth the Lexis diagrams up front using a non-parametric approach, and then extract features of interest from the de-noised data [6, 7, 33, 52–54]. For example, estimates of age-specific period slopes from the smoothed data can be compared to Local Drifts from the Multiplex Analysis. Consistency between the two approaches would bolster conclusions from the model.

Comparative analysis using purely descriptive approaches is time consuming and labor intensive. Our new methods provide a comprehensive, coherent, and reproducible method for small $G$ problems. This covers many outstanding questions in cancer surveillance research. Our essential R code, sample data, and vignettes are freely available.

## Conclusions

It is now possible to evaluate whether estimable functions (EF) from stratified age-period-cohort models are essentially equal, parallel, or distinct. These relationships reflect the presence or absence of proportionality across the strata, conditional on age, period, or birth cohort. Stratum-specific EF that incorporate proportionality are more precise. Comparative Analysis can test a priori hypotheses, or it can identify differences or diversity in proportional relationships between subsets of strata (“pattern heterogeneity”). These new methods can help researchers tackle many outstanding questions in cancer surveillance research.

### Supplementary Information

**Additional file 1.** Online Supplement.

## Data Availability

Our freely available R code is available from the corresponding author upon request. The datasets used in the current study are publicly available through the SEER Program. See https://seer.cancer.gov/data/ for details.
