# Supplementary material for: Comparative age-period-cohort analysis
Source: BMC Med Res Methodol. 2023 Oct 18;23:238. doi: 10.1186/s12874-023-02039-8 (PMC10585891; doi:10.1186/s12874-023-02039-8)
Supplement: Supplementary file 1 — Additional file 1. Online Supplement. [file 12874_2023_2039_MOESM1_ESM.docx]

**Online Supplement**

**1. Cancer Incidence Data**

We extracted cancer incidence data using SEER*Stat software^1^ and the following codes:

SEER*Stat 8 Site and Morphology codes to classify cancer sites.

| **Site** | **Selection Statement** |
| --- | --- |
| Meningioma | {Site and Morphology.Primary Site - labeled} = 'C70.0-Cerebral meninges','C70.1-Spinal meninges','C70.9-Meninges, NOS' |
| Multiple myeloma | {Site and Morphology.Site recode ICD-O-3/WHO 2008} = 'Myeloma' AND {Site and Morphology.ICD-O-3 Hist/behavior, labeled} = '9732/3: Plasma cell (multiple) myeloma' |
|  |  |
| Melanoma | {Site and Morphology.Behavior code ICD-O-3} = 'In situ'  AND {Site and Morphology.Primary Site - labeled} = 'C44.0-Skin of lip, NOS','C44.1-Eyelid','C44.2-External ear','C44.3-Skin other/unspec parts of face','C44.4-Skin of scalp and neck','C44.5-Skin of trunk','C44.6-Skin of upper limb and shoulder','C44.7-Skin of lower limb and hip','C44.8-Overlapping lesion of skin','C44.9-Skin, NOS' |

Note: In situ = ICD-O-3 Histology/behavior (8720-8799)/2

For meningioma^2^ we analyzed 25 two-year age groups from 35 – 36 through 83 – 84 and 7 two-year calendar periods from 2004 – 2005 through 2016 – 2017 spanning 31 nominal birth cohorts centered on birth years 1921, 1923, …, 1981. Data from SEER-13.

For myeloma we analyzed 22 two-year age groups from 41 – 42 through 83 – 84 and 13 two-year calendar periods from 1993 – 1994 through 2017 – 2018 spanning 34 nominal birth cohorts centered on birth years 1910, 1912, …, 1976. Data from SEER-13.

For melanoma^3^ we analyzed 15 five-year age groups 15 – 19 through 85 – 89 and 7 five-year calendar periods 1984 – 1988 through 2014 – 2018 spanning 21 nominal birth cohorts centered on birth years 1899, 1904, …, 1999. Data from SEER-9, race code “white”.

**2. Computer Programs**

*Hypothesis-Based Approach*

We implemented the models described in Sections 3.2.1 – 3.2.6 in MATLAB^4^ version 2023a and R^5^. Our method is a simple case of a Constrained Coefficients GLIM Estimator.

Under N-PH, the design matrix for the joint fit includes a separate set of columns for all APC parameters including each stratum’s trend parameters (Table 1, Row B) and deviations (Table 1, Rows C – E). Under PH-L, there is a *single* column for the shared longitudinal age trend $\left( \alpha_{L}+\gamma_{L} \right)$ and a single set of columns for the shared period and age deviations $\tilde{\pi}$ and $\tilde{\alpha}$, respectively (Table 1, Rows D and E). Under PH-T, the shared columns correspond to the common Net Drift $\left( \pi_{L}+\gamma_{L} \right)$ and the common period and cohort deviations $\tilde{\pi}$ and $\tilde{\gamma}$, respectively. Under PH-X, the shared columns correspond to the common cross-sectional age trend $\left( \alpha_{L}-\gamma_{L} \right)$ and the common age and cohort deviations $\tilde{\alpha}$ and $\tilde{\gamma}$, respectively. Because the cohort deviations are all equal, differences between the Fitted Cohort Patterns (Table 1, Row I) vary linearly and are estimated with increased precision. Under PH-A, all of the columns are shared except for the intercepts.

*Exploratory Approach*

We implemented Algorithm 1 in MATLAB^4^. Analyses of four $50\times27$ strata require 30 seconds on a 2020 Apple M1 Max processor with 64 GB of RAM and no parallelization. For eight $50\times27$ strata (4140 partitions), computation time was 25 minutes, or 5 minutes when Step 1 was parallelized using parfor-loops. We expect this performance can be improved over time, through code optimization and by running the programs on next-generation processors.

1. Surveillance E, and End Results (SEER) Program Surveillance, Epidemiology, and End Results (SEER) Program (<www.seer.cancer.gov>) SEER*Stat Database 8.4.0.1: Incidence - SEER Research Data Plus, 13 Registries, Nov 2021 Sub (1975-2019) - Linked To County Attributes - Time Dependent (1990-2019) Income/Rurality, 1969-2020 Counties, National Cancer Institute, DCCPS, Surveillance Research Program, released April 2022, based on the November 2021 submission. 2021.

2. Bhala SS, D.R.; Kennerley, V; Petkov, V.I.; Rosenberg, P.S.; Best, A.F. Incidence of Benign Meningiomas in the United States: Current and Future Trends. *JNCI Cancer Spectrum* 2021; 5.

3. Olsen CM, Pandeya N, Rosenberg PS and Whiteman DC. Incidence of in Situ vs Invasive Melanoma: Testing the "Obligate Precursor" Hypothesis. *Journal of the National Cancer Institute* 2022; 114: 1364-1370. DOI: 10.1093/jnci/djac138.

4. The MathWorks I. MATLAB: The Language of Technical Computing. 2023a ed. Natick, MA: The MathWorks, Inc., 2023.

5. The R Project for Statistical Computing. R Version 4.3.1, 2023.
